# Prenatal diagnosis of anomalous left brachiocephalic vein courses using high-definition flow render mode and spatiotemporal image correlation

**DOI:** 10.1186/s12947-022-00285-2

**Published:** 2022-06-28

**Authors:** Tian-gang Li, Bin Ma, Ping-an Qi

**Affiliations:** 1 Department of Ultrasound Diagnosis, Gansu Provincial Maternity and Child-care Hospital, Lanzhou, 730050 Gansu Province People’s Republic of China; 2 Gansu Provincial Ultrasound Imaging Clinical Medicine Research Center, Lanzhou, 730050 Gansu Province People’s Republic of China

**Keywords:** Prenatal diagnosis, Fetus, High-definition flow, Three-dimensional ultrasonography, Spatiotemporal image correlation, Anomalous left brachiocephalic vein courses

## Abstract

**Background:**

This study aimed to examine the clinical value of high-definition (HD) flow render mode and spatiotemporal image correlation (STIC) to diagnose anomalous left brachiocephalic vein (LBCV) courses in fetuses.

**Methods and results:**

Seventeen cases of anomalous LBCV courses were diagnosed using two-dimensional (2D), HD-flow, and HD-flow combined with STIC images and retrospectively analyzed to examine the significance of using HD-flow combined with STIC technology in the diagnosis of anomalous LBCV courses.

**Conclusions:**

HD-flow combined with STIC technology can help in the diagnosis of anomalous fetal LBCV courses, and this technique has important clinical value.

## Background

Anomalous left brachiocephalic vein (LBCV) courses in fetuses are very rare, and there is no report of its incidence before delivery. In the literature, the postnatal incidence ranged from 0.2 to 1.7% [1, 2]. Normally, the left jugular vein and left subclavian vein converge on the LBCV, which inclines to the right in the site above the aortic arch and merges with the right brachiocephalic vein into the right superior vena cava (RSVC). Anomalous LBCV courses are classified as retroesophageal, intrathymic, and subaortic. A retroesophageal LBCV may cause mild esophageal compression, but it is asymptomatic [1]. Intrathymic and subaortic LBCV may be normal variations of fetal veins, but most of the fetuses may have abnormal heart structures [3]. On discovery of anomalous LBCV courses in fetuses, the pediatric or cardiac surgery expert should be informed in advance because an anomalous LBCV course may increase procedural difficulty or cause vascular damage during the midline thoracic or cardiac surgery [4]. Fetal echocardiography can diagnose most anomalous LBCV courses and provide accurate information for a prenatal consultation. High-definition (HD) flow combined with spatiotemporal image correlation (STIC) technology can demonstrate the three-dimensional (3D) structure of the fetal heart, aorta, and vein and can display the connection and adjacency between blood vessels to increase diagnostic confidence. This study summarized cases of anomalous LBCV courses diagnosed by HD-flow combined with STIC technology and aimed to provide a reliable basis for the clinical diagnosis of anomalous LBCV courses before delivery.

## Methods

### Study subjects

This retrospective study analyzed ultrasound and clinical data of 17 cases of anomalous LBCV courses diagnosed by fetal echocardiography in our hospital between July 2017 and June 2021.

### Instruments and methods

Images were obtained with an eM6C (2.0–5.0 MHz) transducer from a Voluson E10 US system (GE Healthcare, Zipf, Austria). First, conventional two-dimensional (2D) ultrasonography was used to determine the presence of structural abnormalities in the fetus. Subsequently, fetal echocardiography was performed by an expert sonographer to determine whether the intracardiac structure of the fetus is abnormal. HD-flow combined with STIC technology was used to visualize the LBCV and great blood vessels to determine the anomalous course of the LBCV.

Prenatal ultrasound results were determined by two chief physicians engaged in fetal echocardiography, and imaging characteristics of the anomalous LBCV courses were analyzed. Fetal cardiac 2D, HD-flow, and HD-flow combined with STIC technology were respectively retrieved for analysis. All newborns were examined by ultrasonography or computed tomography angiography (CTA) after delivery.

**Fig. 1:**
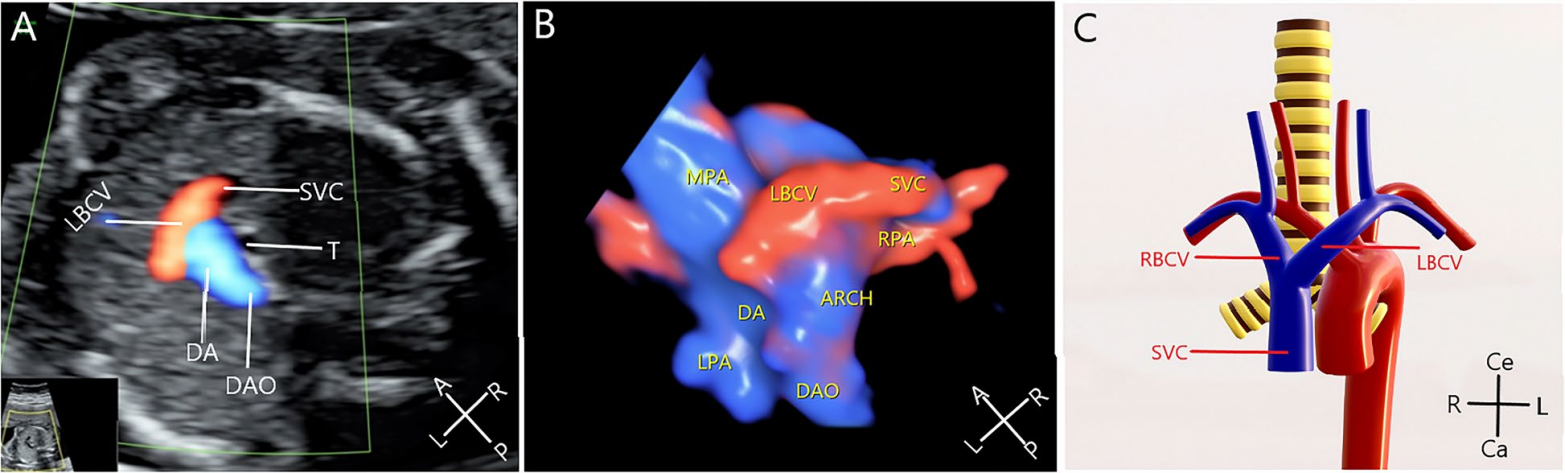
Images of the normal LBCV using two-dimensional (2D) and three-dimensional (3D) ultrasonography. **A** Color blood flow showing that the LBCV merges into the RSVC. **B** LBCV and RSVC are shown using HD-flow render mode and STIC. **C** Normal structural presentation of the LBCV and RSVC (anterior view). SVC, superior vena cava; DA, ductus arteriosus; DAO, descending aorta; RSVC, right superior vena cava; SP, spine, T, trachea; LBCV, left brachiocephalic vein; L, left; R, right; A, abdominal; P, spinal; Ce, cephalic; Ca, caudal

Normal LBCV: The HD-flow color blood flow in the three-vessel tracheal (3VT) view of the fetus shows that the LBCV merges into the RSVC (Fig. 1A). The 3D image clearly shows the LBCV runs over the aortic arch and enters the RSVC (Fig. 1B). The normal structural presentation shows that the LBCV enters the RSVC (Fig. 1C).

**Fig. 2:**
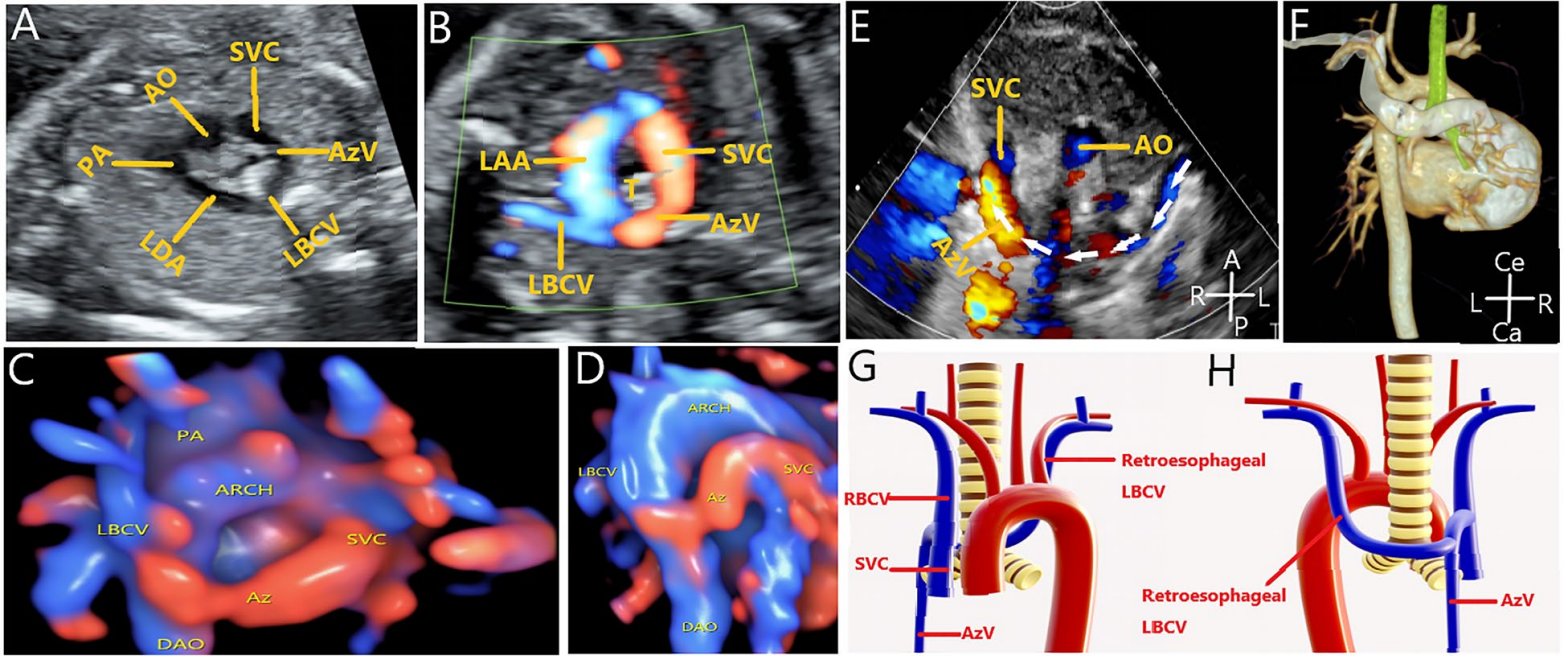
Image of the retroesophageal LBCV. **A** The LBCV runs behind the aorta and flows into the RSVC to form a U-shaped structure. **B** Color blood flow showing the retroesophageal LBCV. **C–D** Retroesophageal LBCV is shown using HD-flow render mode and STIC. **E** Postpartum 2D ultrasound image showing that the LBCV bypasses the esophagus. **F** Postpartum CTA showing that the LBCV bypasses the esophagus and merges into the RSVC, which was consistent with the prenatal image. **G-H** Structural presentation of a retroesophageal LBCV. **G** Anterior view. **H** Posterior view. LBCV, left brachiocephalic vein. AO, aorta; PA, pulmonary artery; SVC, superior vena cava; LDA, left ductus arteriosus; AzV, azygos vein; LAA, left aortic arch; ARCH, aortic arch; T, trachea; DAO, descending aorta; RBCV, right brachiocephalic vein; L, left; R, right; Ce, cephalic; Ca, caudal; A, abdominal; P, spinal

Retroesophageal LBCV: 2D images and HD-flow color blood flow in the 3VT section of the fetus show that transverse abnormal blood vessels run behind the aorta and trachea and then flow into the azygos vein and inflow to the RSVC to form a U-shaped structure (Fig. 2A). Color Doppler ultrasound examination showed the “U”-shaped structure in different colors (Fig. 2B). HD-flow combined with STIC technology clearly shows that the LBCV went around the esophagus and merged into the RSVC (Fig. 2C, D). Postpartum 2D and color Doppler ultrasound images show that the LBCV bypassed the esophagus (Fig. 2E). Postpartum CTA shows that the LBCV bypassed the esophagus and merged into the RSVC, which was consistent with prenatal ultrasound images (Fig. 2F). The structural presentation of retroesophageal LBCV shows that the LBCV enters the RSVC (Fig. 2G, H).

**Fig. 3:**
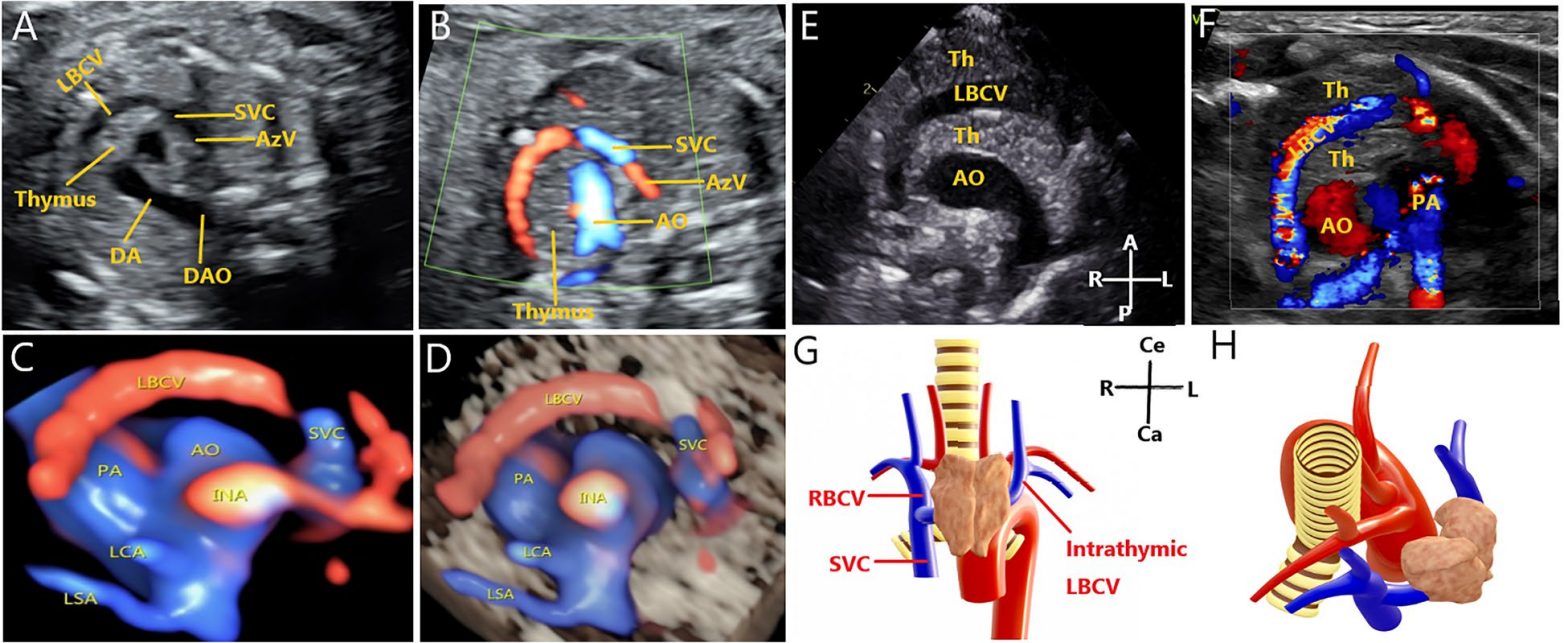
Images of the intrathymic LBCV using two-dimensional (2D) and three-dimensional ultrasonography. **A** 2D ultrasound image showing intrathymic LBCV. **B** Color Doppler blood flow demonstrating intrathymic LBCV. **C-D** HD-flow render mode and spatiotemporal image correlation showing that the LBCV passes through the thymus. **E–F** Postpartum 2D and color Doppler ultrasound images showing that the LBCV runs in the thymus, which demonstrates good consistency with the prenatal ultrasound image. **G-H** Structural presentation of intrathymic LBCV. **G** Anterior view. **H** Superior view. AO, aorta; PA, pulmonary artery; SVC, superior vena cava; DA, ductus arteriosus; AzV, azygos vein; DAO, descending aorta; LBCV, left brachiocephalic vein; Th, thymus; LSA, left subclavian artery; RBCV, right brachiocephalic vein; L, left; R, right; A, abdominal; P, spinal; Ce, cephalic; Ca, caudal

Intrathymic LBCV: 2D and color blood flow images were used to show the LBCV running in the thymus (Fig. 3A, B). HD-flow combined with STIC technology clearly shows the intrathymic LBCV (Fig. 3C, D). Postpartum 2D and color Doppler ultrasound images show that the LBCV runs in the thymus (Fig. 3E, F). The structural presentation shows that the intrathymic LBCV enters the RSVC (Fig. 3G, H).

**Fig. 4:**
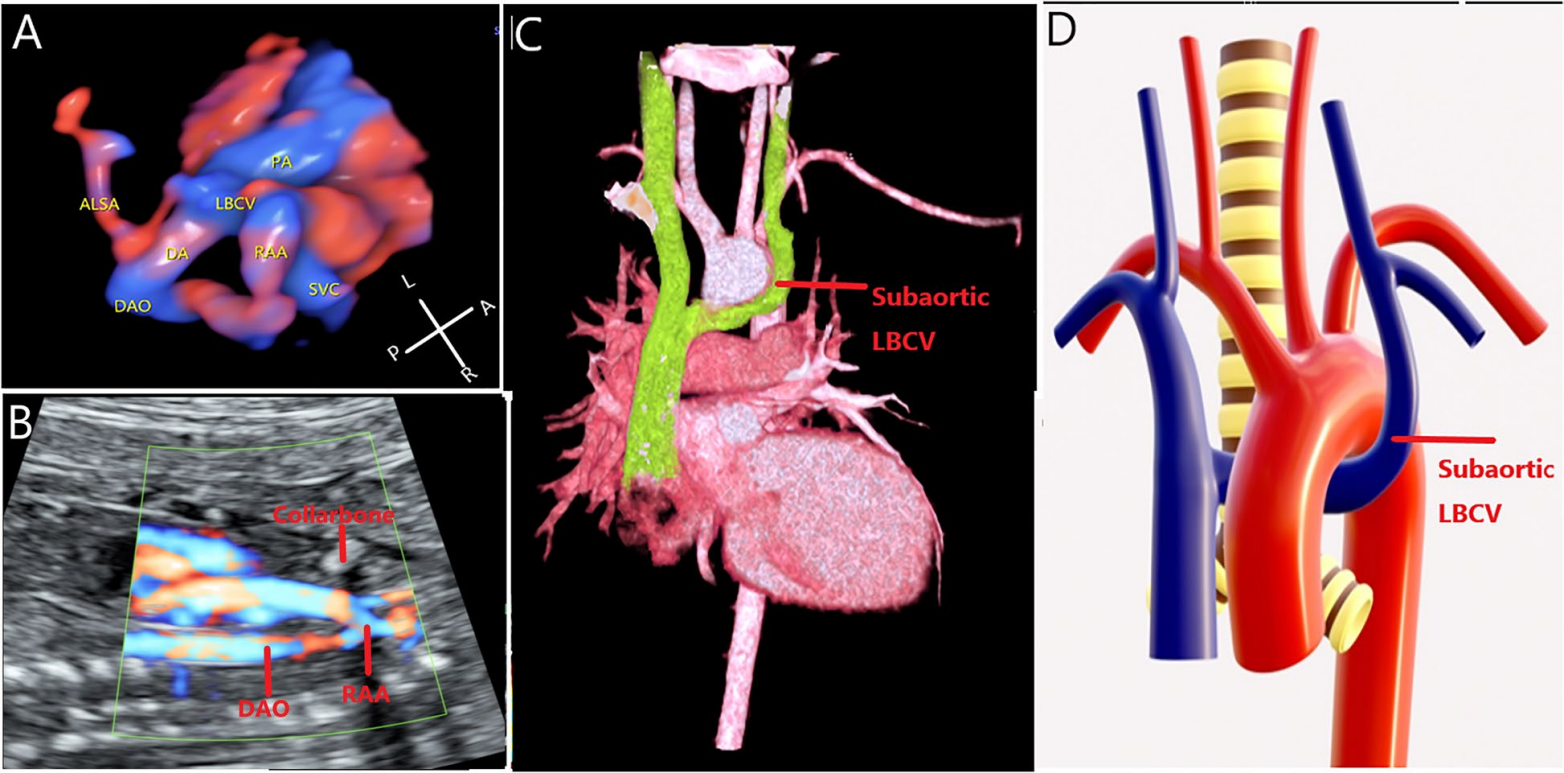
Prenatal and postpartum images of the subaortic LBCV. **A** Three-dimensional high-definition flow render mode and STIC are used to show the subaortic LBCV in a fetus. **B** Cervical aortic arch corresponds to the level of the collarbone. **C** Postpartum CTA showing that the subaortic LBCV, which was consistent with the prenatal image. **D** Structural presentation of subaortic LBCV. LBCV, left brachiocephalic vein; RAA, right aortic arch; PA, pulmonary artery; SVC, superior vena cava; DA, ductus arteriosus; DAO, descending aorta; LBCV, left brachiocephalic vein; L, left; R, right; A, abdominal; P, spinal

Subaortic LBCV: HD-flow combined with STIC technology can clearly show that the LBCV runs beneath the aortic arch (Fig. 4A). Cervical aortic arch corresponds to the level of the collarbone (Fig. 4B). Postpartum CTA shows the subaortic LBCV (Fig. 4C). The structural presentation shows that the subaortic LBCV enters the RSVC (Fig. 4D).

## Results

Seventeen cases were all singletons, and there were four cases of retroesophageal LBCV, including two cases of isolated retroesophageal LBCV and two cases with structural malformations. Three cases of intrathymic LBCV were noted, including one isolated case and two cases with structural abnormalities. Ten cases of subaortic LBCV were identified, including two isolated cases and eight cases with intracardiac or extracardiac structural malformations. The HD-flow and HD-flow combined with STIC shows that all LVBC drain to the SVC and there was no dilated LBCV or absent LBCV in the study.

Retroesophageal LBCV (*n* = 4) and intrathymic LBCV (*n* = 3) were diagnosed in the second trimester. Two cases of subaortic LBCV were diagnosed in the third trimester. All the cases were identified accurately by using 2D images, HD-flow, and 3D images with STIC in the second trimester. The clinical characteristics of seventeen cases of anomalous left brachiocephalic vein courses was showed in Table 1. 

**Table 1:** Clinical characteristics of seventeen cases of anomalous left brachiocephalic vein courses

Type	Age	Gestational week for scan	Gestational week of delivery	Combined deformation	Outcome	Mode of delivery
Retroesophageal LBCV	34	27	N/A	PTA、OM、VSD、DH、He、SUA、AF	Termination of pregnancy	N/A
Retroesophageal LBCV	24	24	38^+6^W	PLSVC	Good	Vaginal
Retroesophageal LBCV	36	24	39^+ 5^ W	Isolated	Good	Cesarean
Retroesophageal LBCV	25	23	40^+ 2^W	Isolated	Good	Vaginal
Intrathymic LBCV	25	26	39^+ 4^ W	RAA-MB	Good	Vaginal
Intrathymic LBCV	31	23	40^+ 5^ W	Isolated	Good	Vaginal
Intrathymic LBCV	31	25	N/A	PTA	Termination of pregnancy	N/A
Subaortic LBCV	21	24	39^+ 2^ W	RAA with ALSA	Good	Vaginal
Subaortic LBCV	31	21	38^+6^W	TOF	Surgical treatment after birth	Vaginal
Subaortic LBCV	32	28	N/A	TOF、 Absence of DA	Termination of pregnancy	N/A
Subaortic LBCV	26	26	39^+0^W	RAA with ALSA, CAA	Good	Cesarean
Subaortic LBCV	27	24	40^+ 1^ W	Isolated	Good	Vaginal
Subaortic LBCV	28	23	36^+3^W	RAA with ALSA, CAA	Good	Vaginal
Subaortic LBCV	28	23	39^+3^W	RAA with ALSA	Good	Vaginal
Subaortic LBCV	31	25	38^+ 5^ W	RAA with ALSA, CAA	Good	Cesarean
Subaortic LBCV	27	24	40^+ 2^ W	RAA with ALSA	Good	Vaginal
Subaortic LBCV	28	24	39^+5^W	Isolated	Good	Cesarean

*LBCV* Left brachiocephalic vein, *PTA* Persistent truncus arteriosus, *OM* omphalocele, *VSD* Ventricular septal defect, *DH* Diaphragmatic Hernia, *He* Hemivertebra, *SUA* Single umbilical artery, *AF* Ascitic fluid, *PLSVC* Perpetual left superior vena cava, *RAA-MB* Right aortic arch with mirror branch, RAA with ALSA Right aortic arch with aberrant left subclavian artery, *TOF* Tetralogy of fallot, Absence of ductus arteriosus, *CAA* Cervical aortic arch

## Discussion

Although anomalous LBCV courses are very rare before delivery, they have been reported given the widespread use of fetal echocardiography [4–9]. The 3VT view is the most important view for the diagnosis of anomalous LBCV courses because the 3VT view can show fetal blood vessels, cardiac vessels, and the thymus [3, 10, 11]. Normally, the 3VT view is slightly deflected to the head of the fetus, and the LBCV can be displayed above the aortic arch and behind the thymus [5]. In previous studies, fetal retroesophageal LBCV had been reported [4, 7, 8]. Very few studies have also used STIC technology to describe retroesophageal LBCV in fetuses^7^. Prenatal diagnosis of subaortic LBCV and intrathymic LBCV were rarely reported, mainly in children or adults with CT and magnetic resonance imaging (MRI) findings [1, 12]. This study shows that the HD-flow combined with STIC can help show the adjacent relationship between the LBCV and aortic arch, and it has high clinical application value for accurately diagnosing the anomalous LBCV courses in fetuses.

The retroesophageal LBCV in the 3VT view can show that the transverse LBCV runs behind the aorta and trachea and then enters the RSVC through the azygous vein. Retroesophageal LBCV can form a “U”-shaped structure with the aortic arch. However, the color Doppler ultrasound image shows that the “U”-shaped structure has a different color, which needs to be distinguished from the right aortic arch (RAA). The RAA shows a U-shaped vascular ring of the same color. In addition, spectral Doppler ultrasonography can be used to observe the low-velocity venous blood flow spectrum instead of the arterial spectrum with retroesophageal LBCV. This study shows that HD-flow combined with STIC technology can intuitively display the retroesophageal LBCV finally flowing into the RSVC through the azygous vein.

When the LBCV runs inside the thymus, the LBCV is in a more forward position, partly curved, and merges into the RSVC from left to right. Ultrasound images showed an increase in the distance between the LBCV and the left brachiocephalic artery (LBCA) in the 3VT view. It is defined by the presence of thymic tissues between the LBCV and LBCA. A few studies have reported about the fetal intrathymic LBCV. Some studies have shown that the intrathymic LBCV may be more common in neonates. The main reason is that most intrathymic LBCV cases are isolated and easily missed. This study shows that the use of HD-flow combined with STIC can display the relationship between the LBCV and the thymus, and it can assist in diagnosing intrathymic LBCV in fetuses. When thymic surgery is required after birth, injuries to the LBCV and adverse outcomes should be monitored.

Subaortic LBCV is also known as LBCV in a low position. In this case, the LBCV runs under the aortic arch and enters the RSVC. This type is also rarely reported before delivery. Subaortic LBCV is often associated with conus trunk and aortic arch malformation. With the gradual application of imaging techniques such as CTA and MRI in clinical examinations, the subaortic LBCV is more commonly found in children and adults. Because the subaortic LBCV is often missed, its incidence may be underestimated. In our study, only three cases of isolated subaortic LBCV were identified, which was lower than the cases with cardiac malformations. The most common cardiac malformations of subaortic LBCV are RAA with aberrant left subclavian artery (ALSA) and cervical aortic arch (CAA). The RAA with ALSA and CAA can closely related to 22q11.2 chromosome microdeletion. Therefore, in fetuses having an RAA with ALSA or CAA, subaortic LBCV should be excluded and chromosomal copy number variants should be detected. In addition, subaortic LBCV, right pulmonary artery, and trachea can be displayed under the aortic arch in the long-axis view of the aortic arch, which is important to determine subaortic LBCV, but it is not easy to show in every case.

After anomalous LBCV courses are confirmed prenatally, comprehensive prenatal ultrasonography and fetal echocardiography are necessary to detect fetal abnormalities in extracardiac or intracardiac structures. If necessary, chromosome analysis is required. A study reported that anomalous LBCV courses are not clearly related to chromosomal abnormalities [13]. Most cases of isolated anomalous LBCV courses are not associated with the presence of other malformations, and in these cases, chromosomal alterations are not usually present [3]. Isolated anomalous LBCV courses have a better clinical prognosis because of less compression of the trachea or esophagus, which can be considered as a normal variant. In a fetus with an anomalous LBCV course, the cardiologist should be reminded about this anomaly because it may affect the centerline or heart surgery method [14]. HD-flow render mode and STIC can more clearly and intuitively display the adjacent relationship between the aortic arch and the LBCV, and it can better display the entire drainage route of the LBCV than 2D image. With this method, we cannot only explain the anatomical variation of the anomalous LBCV course to the pregnant woman, but also mention that it has certain guiding significance for making decisions about cardiac malformation surgery and potential cardiac interventional surgery in the future. In this study, although most fetuses with anomalous LBCV courses can be diagnosed by 3VT view combined with color blood flow before childbirth, HD-flow combined with STIC can dynamically better display the anomalous LBCV course and make a clear classification than 2D image. In addition, the STIC image of anomalous LBCV courses is highly consistent with the postpartum ultrasound images or CTA. HD-flow combined with STIC presents an intuitive 3D structure of the anomalous LBCV courses, which is helpful for the accurate diagnosis of the anomalous LBCV course before delivery.

Obtaining 3D images of the anomalous LBCV course in fetuses may be affected by factors such as fetal movement. Therefore, images may be obtained while the fetus is in a quiet state. In addition, sufficient patience is necessary to obtain a satisfactory STIC image of the anomalous LBCV course in fetuses. Finally, due to the small sample size in this study, a larger-scale prospective evaluation and analysis should be carried out in the future to obtain more objective indicators and information to guide clinical practice.

## Data Availability

The data and material in the current study are available from the corresponding author on reasonable request.
